# Traitement des Suppurations pelviennes. De l'Hystérectomie abdominale totale. Traitement chirurgical des Rétrodéviations utérines. Du meilleur Mode de Fermeture de la Paroi abdominale

**Published:** 1897-09

**Authors:** 


**Traitement des Suppurations pelviennes. De l'Hystérectomie abdominale totale. Traitement chirurgical des Rétro-déviations utérines. Du meilleur Mode de Fermeture de la Paroi abdominale.** Par EUGÈNE DOYEN. Pp. 78. Paris: Institut international de Bibliographie scientifique. 1896.

M. Doyen is an original thinker, and must be regarded as one of the leaders in abdominal surgery. His methods, which differ in many details from those usually adopted, are here clearly set forth and supplemented by some useful illustrations. We have before had reason to speak well of the way in which these *brochures* are prepared, and the present collection of essays is quite equal to its predecessors.

